# Polypharmacy and anticholinergic burden are common but not independently associated with outcomes after emergency laparotomy in older adults

**DOI:** 10.1007/s00423-026-04060-z

**Published:** 2026-04-21

**Authors:** Randeep S. Heer, Amit K. J. Mandal, Vadir Baktash, Fahad Javed, Rebecca Atkins, Hannah Binns, Sharmistha Gupta, Ravi Vissapragada, Constantinos G. Missouris, Henry D. De’Ath

**Affiliations:** 1 Departments of Medicine and Cardiology, Wexham Park Hospital, Wexham Street, Slough, SL2 4HL UK; 2 Departments of Medicine and Elderly Care, Frimley Park Hospital, Portsmouth Road, Frimley, Camberley GU16 7UJ UK; 3 Departments of Anaesthesia and Critical Care, Wexham Park Hospital, Wexham Street, Slough, SL2 4HL UK; 4 Discipline of Surgery, College of Medicine and Public Health, Flinders University, Adelaide, Australia; 5 Academic Department of Clinical Cardiology, University of Nicosia Medical School, Nicosia, Cyprus; 6 Department of Upper Gastrointestinal Surgery, Frimley Park Hospital, Portsmouth Road, Frimley, Camberley GU16 7UJ UK

**Keywords:** Polypharmacy, Anticholinergic burden, Laparotomy, Outcomes, Frailty, Older adults

## Abstract

**Purpose:**

Anticholinergic burden (ACB) is an accumulative effect of medications with individual anticholinergic properties. ACB arising from polypharmacy is common in older adults and has been linked to poorer outcomes in a variety of clinical contexts. With more older patients undergoing surgery, there is greater exposure to higher risk individuals with polypharmacy and increased ACB, although the significance of these postoperatively is unknown. The purpose of the study was to determine the frequency of polypharmacy and accompanying ACB in older patients undergoing laparotomy and to investigate their effect on outcomes.

**Methods:**

An ambispective, observational cohort study was conducted across two UK hospitals and included patients aged ≥ 65 undergoing emergency laparotomy, as per National Emergency Laparotomy Audit (NELA) criteria, over a 2-year period. Patients with polypharmacy (≥ 5 drugs) or high ACB (ACB score ≥ 3) were identified. Primary outcome was mortality up to 1 year and secondary outcomes included postoperative complications, functional and cognitive decline, intensive care unit (ICU) admission, length of hospital stay and readmission within 30-days.

**Results:**

A total of 144 patients were included (median age 76, 54% female) of which 42% had polypharmacy and 17% had high ACB. There was no significant difference in mortality up to 1 year following surgery between those with or without polypharmacy, nor those with or without significant ACB. Patients with polypharmacy had longer hospital stays (15 vs. 12 days, *p* = 0.025). There were no significant differences demonstrated for other secondary outcomes.

**Conclusions:**

Polypharmacy and high ACB are common in older adults undergoing emergency laparotomy but neither appeared to influence adverse outcomes apart from increased length of hospitalisation. Polypharmacy and ACB may simply be surrogate markers for age, frailty and comorbidity burden. The relationship should be further explored in larger, well-designed studies.

**Supplementary Information:**

The online version contains supplementary material available at 10.1007/s00423-026-04060-z.

## Introduction

The most widely accepted definition of polypharmacy is prescription of five or more medications [1, 2]. The high prevalence of polypharmacy, which affects one quarter of 69-year-olds in the United Kingdom (UK), is a consequence of an ageing population with an increasing rate of multimorbidity [3]. Polypharmacy is associated with negative outcomes, including adverse drug-related events, non-adherence, disability and mortality [4, 5].

Anticholinergic burden (ACB) is an accumulative effect of medications with individual anticholinergic properties. ACB is emerging as a risk factor for a myriad of adverse events that disproportionately affect older adults including autonomic dysfunction, falls, dry mouth and eyes, delirium, and progression of neurodegenerative disease [6]. Numerous frequently used drugs exert strong anticholinergic effects with undesirable impact across many physiological systems reflecting the wide distribution of muscarinic receptors in both peripheral and central nervous systems. Many of these medications cross the blood brain barrier and influence the central nervous system directly and this is more pronounced in older adults where age-related changes in the blood brain barrier disrupt its function [7]. Furthermore, commonly prescribed drugs to treat chronic conditions such as heart disease, hypertension, depression, urinary incontinence, pain and allergies individually carry weak anticholinergic properties, but in combination confer significant ACB [8–10].

Currently, there is no consensus on how to calculate ACB. Multiple tools have been developed including the Anticholinergic Burden Scale, Anticholinergic Drug Scale, Anticholinergic Cognitive Burden Scale and Anticholinergic Risk Scale (ARS) with different tools using varied resources and properties to calculate ACB with slight variability [11]. Effects of ACB are dependent both on exposure time and dose of medication. In general, each drug is given an ACB score of 0–3 depending on the extent of the drug’s anticholinergic effects with a total score for a patient of 3 or greater considered clinically significant [11]. Polypharmacy further predisposes to higher ACB, and higher scores are proportionate to increased adverse outcomes, including fall, functional decline, delirium and death [12].

Reflective of the ageing population, there has been an exponential rise in patients undergoing surgical procedures. In an analysis of hospital records in England, the number of people aged 75 years or older undergoing surgery increased from 544,998 in 1999 to 1,012,517 in 2015, whilst the average age of surgical patients has increased from 47.5 years in 1999 to 54.2 years in 2015 [13]. The National Emergency Laparotomy Audit (NELA) is a collaborative initiative by the Royal Colleges in the UK designed to record highly reliable data to facilitate improvement in patient outcomes after laparotomy [14]. Historical data analyses confirm that patients aged ≥ 65 years have worse clinical outcomes compared to younger counterparts, including lengthier hospitalisation and higher 30-day and 90-day mortality rates [15, 16]. In addition, there is an association between frailty, which is known to escalate with age, and increased 90-day mortality following emergency laparotomy [17]. Emphasis is therefore laid upon the importance of specialist perioperative input from medical teams in the multidisciplinary management and pharmacological optimisation of older patients in the surgical environment.

To date, the evidence surrounding polypharmacy and associated ACB on post-operative outcomes remains conflicting. In particular, data are limited in older adults after emergency major abdominal surgery. This study aimed to measure the incidence of polypharmacy and significant ACB among older patients undergoing emergency laparotomy and to explore any relationship with adverse outcomes.

## Materials and methods

### Study design and setting

An ambispective, observational cohort study was performed at two district general hospitals in the UK. Sites searched medical records to identify patients aged 65 years and above who underwent emergency surgery according to NELA criteria between 1 st January 2020 to 1 st March 2022 [18]. This included patients who underwent open, laparoscopic, or laparoscopically assisted procedures (Supplementary File 1). Patients with a diagnosis of Covid-19 were excluded due to its potential strong confounding effect on the study’s outcomes at the time of data collection.

### Data collection

For eligible patients, data collected included age, sex, ASA grade, Rockwood Clinical Frailty Score (CFS), comorbidities according to the Charlson Comorbidity Index and medications prescribed prior to admission (collated using pharmacist validated records) [19]. CFS was grouped into three clinically meaningful categories (fit (0–2), mild frailty (3–4), and moderate-to-severe frailty (5–7)) to address sparse outcome events in low score groups. For those without documented CFS, pre- and postoperative CFS was calculated retrospectively. Polypharmacy was deemed present if a patient was prescribed five or more oral medications prior to admission [2]. Insulin was excluded from this count due to its vital essential life-sustaining role and inhalers and topical agents were excluded due to their minimal likelihood of systemic effects relevant to this study at common therapeutic doses. ACB for each patient was calculated using an online calculator which combined the anticholinergic cognitive burden scale and the German anticholinergic burden scale [20–22]. The scores assigned to each medication are available in supplementary file 1. Drugs with possible ACB were scored 1 whilst drugs with definite ACB were scored 2 or 3. ACB was deemed significant if the total score for a patient was 3 or more. The date and type of surgery performed were also recorded. Patient outcomes were assessed up to 1 year after the date of surgery.

### Outcomes

The primary outcome was mortality, assessed at various timepoints including as an inpatient, at 30 days, 90 days, 6 months and 1 year following surgery. Secondary outcomes included inpatient surgical complications classified according to the Clavien Dindo score [23], post-operative ICU admission (planned or unplanned), length of hospital stay and readmission to hospital within 30-days. Post-operative delirium was deemed present if inpatient medical notes documented new onset confusion or delirium, or worsened confusion compared to baseline if there was pre-existing cognitive impairment. Patients were classified as being discharged with an increase in care needs if medical notes documented a discharge destination with higher care levels (such as residential home, nursing home or rehabilitation centre), requiring carers (either formal or informal), or discharged with assistive equipment provided by an inpatient occupational therapist or physiotherapist. Those who died as an inpatient were excluded from this calculation.

### Statistical analysis

Normal—quantile plots were used to test for normality. Continuous data are presented as median (interquartile range) and categorical data as absolute number (percentage). Continuous data were analysed using the Mann-Whitney U test. Chi-square test for trend was used to compare categorical data. A two tailed p value of < 0.05 was considered significant. These analyses were performed using GraphPad Prism version 10 (GraphPad Software, Boston, MA, USA). A multivariable logistic regression model was used to examine the association between polypharmacy and significant ACB with one year mortality. Polypharmacy and significant ACB were entered as the main explanatory variables. The model was adjusted for age, sex, Charlson Comorbidity Index score, frailty score and type of surgery, which were selected a priori as clinically relevant confounders. ASA was not included to minimise collinearity and to preserve model stability, given the number of outcome events. This analysis was carried out on RStudio. Results are presented as adjusted odds ratios with ninety-five per cent confidence intervals.

### Ethics

The study complied with the Declaration of Helsinki and was approved by the local Audit and Quality Improvement Department (reference FH509).

## Results

### Study demographics

A total of 144 patients were included during the study time frame (Table 1). The median age was 76 years, with a slightly greater proportion of females (54%). Patients were generally frail with an ASA grade of 3 and Charlson Comorbidity Index (CCI) of 4. The most common co-morbidities were hypertension and solid organ malignancy (53% and 23% of patients respectively).

**Table 1:** Baseline characteristics

	Overall	Polypharmacy			ACB score ≥ 3		
		Yes	No	*p*	Yes	No	*p*
***n***	144	61	83		24	120	
**Age**, *years*,* median (IQR)*	76 (83 − 70)	78 (84 − 72)	74 (80 − 69)	**0.026**	74 (80.75–71.25)	77 (83 − 70)	0.357
**Sex** (M: F)	66:78	29:32	37:46	0.738	6:18	60:60	**0.023**
**Clinical Frailty Scale**							
Fit	32	7 (11)	25 (30)	**0.009**	3 (13)	29 (24)	0.286
Mild frailty	82	34 (56)	48 (58)	0.865	13 (54)	69 (58)	0.823
Moderate-to-severe frailty	30	20 (33)	10 (12)	**0.003**	8 (33)	22 (18)	0.106
**ASA grade**, *median (IQR)*	3 (3 − 2)	3 (4 − 3)	3 (3 − 2)	**< 0.001**	3 (3 − 2)	3 (3 − 2)	0.644
***Co-morbidities***							
CRD	24 (17)	15 (25)	9 (11)	**0.041**	7 (29)	17 (14)	0.128
IHD	12 (8)	8 (13)	4 (5)	0.124	1 (4)	11 (9)	0.691
Heart failure	4 (3)	4 (7)	0	**0.030**	2 (8)	2 (2)	0.129
Hypertension	76 (53)	38 (62)	38 (46)	0.063	14 (58)	62 (52)	0.656
PVD	9 (6)	6 (10)	3 (4)	0.169	2 (8)	7 (6)	0.645
Stroke/TIA	14 (10)	10 (16)	4 (5)	**0.025**	2 (8)	12 (10)	> 0.999
Dementia	4 (3)	2 (3)	2 (2)	> 0.999	1 (4)	3 (3)	0.52
PUD	5 (3)	3 (5)	2 (2)	0.650	1 (4)	4 (3)	> 0.999
Liver disease	8 (6)	4 (7)	4 (5)	> 0.722	2 (8%)	6 (5)	0.620
Diabetes Mellitus	29 (20)	18 (30)	11 (13)	**0.021**	4 (17%)	25 (21)	0.785
CKD	13 (9)	7 (11)	6 (7)	0.395	2 (8)	11 (9)	> 0.999
Leukaemia/lymphoma	4 (3)	2 (3)	2 (2)	> 0.999	2 (8)	2 (2)	0.129
Solid Organ Malignancy	33 (23)	19 (31)	14 (17)	**0.048**	7 (29)	26 (22)	0.432
Connective tissue disease	11 (8)	5 (8)	6 (7)	> 0.999	2 (8)	9 (8)	> 0.999
CCI score, *median (IQR)*	4 (6 − 3)	5 (6 − 4)	4 (5 − 3)	**0.004**	4 (5-3.25.25)	4 (6 − 3)	0.926

Data are presented as absolute number (percentage) unless otherwise stated. CRD=chronic respiratory disease. IHD=ischaemic heart disease. PVD=peripheral vascular disease. PUD=peptic ulcer disease. CCI=Charlson Co-morbidity Index

Of these, 61 patients (42%) met the criteria for polypharmacy. These individuals were significantly older than non-polypharmacy counterparts (median age 78 vs. 74 years respectively), but with no difference between the sexes. However, they were more likely to have higher ASA and CCI scores, and increased rates of respiratory disease, heart failure, cerebrovascular accidents, diabetes and malignancy. Additionally, there was a lower proportion of patients classified as fit on the Rockwood CFS in those with polypharmacy and a greater proportion of those with moderate-to-severe frailty in those with polypharmacy.

We identified 24 patients (17%) with significant ACB score and a higher proportion were female. However, there were no significant differences between the groups with regards to other parameters including frailty, median age and prevalence of comorbidities.

### Operative types

Most operations were performed as a laparotomy with less than 10% undertaken laparoscopically (Table 2). The commonest indication for surgery was small bowel obstruction, and consequently the most frequent surgery adhesiolysis. Thereafter, small bowel resections, Hartmann’s for perforations of the sigmoid colon (in the majority of cases due to diverticular disease), and right hemicolectomies (predominantly due to obstructive and emergency presentations of colonic cancer) were the most frequent procedures. There were no differences in the nature and frequency of surgeries between the polypharmacy and non-polypharmacy groups, nor indeed between the ACB score ≥ 3 group versus those lower.

**Table 2:** Characteristics of operative procedures

	All	Polypharmacy			ACB score ≥ 3		
		Yes	No		Yes	No	
*n*	144	61	83		24	120	
**Operative Approach**							
Laparotomy	134 (93)	56 (92)	78 (94)	0.743	22 (92)	112 (93)	0.673
Laparoscopy	10 (7)	5 (8)	5 (6)		2 (8)	8 (7)	
**Operative** **Procedure**							
Adhesiolysis	30 (21)	15 (25)	15 (18)	0.408	6 (25)	24 (20)	0.587
Small bowel resection	28 (19)	10 (16)	18 (22)	0.524	3 (13)	25 (21)	0.413
Subtotal colectomy	7 (5)	4 (7)	3 (4)	0.457	1 (4)	6 (5)	> 0.999
Hernia repair (abdominal)	10 (7)	4 (7)	6 (7)	> 0.999	1 (4)	9 (8)	> 0.999
Hartmanns	22 (15)	6 (10)	16 (19)	0.161	2 (8)	20 (17)	0.533
Ileostomy formation	13 (9)	7 (11)	6 (7)	0.395	1 (4)	12 (10)	0.6955
Left hemicolectomy	5 (3)	0	5 (6)	0.073	0	5 (4)	0.591
Right hemicolectomy	18 (13)	5 (8)	13 (16)	0.2108	1 (4)	17 (14)	0.3089
Perforated gastric ulcer repair	6 (4)	3 (5)	3 (4)	0.698	0	6 (5)	0.589
Perforated duodenal ulcer repair	3 (2)	0	3 (4)	0.262	0	3 (3)	> 0.999
Colostomy formation	8 (6)	2 (3)	6 (7)	0.4673	2 (8)	6 (5)	0.6205
Enterotomy	2 (1)	1 (2)	1 (1)	> 0.999	0	2 (2)	> 0.999
Not amenable for surgery	3 (2)	2 (3)	1 (1)	0.574	1 (4)	2 (2)	0.433
Other	7 (5)	6 (10)	1 (1)	**0.042**	6 (25)	1 (1)	**< 0.001**

Data are presented as absolute number (percentage) unless otherwise stated. Other includes intestinal perforation repair, intestinal bypass, washout, percutaneous endoscopic gastrostomy retrieval with colonic repair

### Outcomes

The overall mortality of the study cohort within a year was 17%, and 9% of patients died in hospital (Table 3). There was no detectable statistically significant difference in mortality between those with or without polypharmacy, nor those with or without high ACB score at all the studied time points.

**Table 3:** Outcomes

	All	Polypharmacy			ACB score ≥ 3		
		Yes	No	*p*	Yes	No	*p*
*n*	144	61	83		24	120	
***Mortality***							
In hospital	13 (9)	6 (10)	7 (8)	> 0.999	0	13 (11)	0.126
Within 30 days	12 (8)	6 (10)	6 (7)	0.762	0	13 (11)	0.126
Within 90 days	16 (11)	8 (13)	8 (10)	0.595	0	16 (13)	0.074
Within 6 months	18 (13)	9 (15)	9 (11)	0.611	2 (8)	16 (13)	0.738
Within 1 year	25 (17)	11 (18)	14 (17)	> 0.999	3 (13)	22 (18)	0.767
***Clavien dindo score***							
I	46 (32)	16 (26)	30 (36)	0.278	12 (50)	34 (28)	0.052
II	39 (27)	21 (34)	18 (22)	0.128	6 (25)	33 (28)	> 0.999
IIIa	6 (4)	2 (3)	4 (5)	> 0.999	0	6 (5)	0.589
IIIb	4 (3)	1 (2)	3 (4%)	0.637	0	4 (3)	> 0.999
IVa	20 (14)	9 (15)	11 (13)	0.812	5 (21)	15 (13)	0.331
IVb	8 (6)	3 (5)	5 (6)	> 0.999	1 (4)	7 (6)	> 0.999
***Post-operative delirium***	12 (8)	4 (7)	8 (10)	0.5594	1 (4)	11 (9)	0.6908
***Increase in care needs*** [*n* = 131]	36 (27)	17 (31)	19 (25)	0.5525	4 (17)	32 (30)	0.2174
***Post-operative ICU admission***	93 (65)	41 (67)	52 (63)	0.601	12 (50)	81 (68)	0.109
***Length of hospital stay***, *median (IQR)*	13 (22 − 8)	15 (24.5–9.5)	12 (20.25-7.25)	**0.025**	13.50 (23.5–11)	13 (22 − 8)	0.689
***Readmission within 30 days***	16 (11)	9 (15)	7 (8)	0.287	5 (21)	11 (9)	0.146

Data are presented as absolute number (percentage) unless otherwise stated

A total of 123 patients suffered a complication, excluding mortality, defined as Clavien-Dindo score I – IVb. The most common complication grade experienced by patients was I and II (32% and 27% of patients respectively). However, there was no statistically significant difference detected in the incidence of each complication grade between those with or without polypharmacy, nor those with or without a significant ACB score.

In the overall study cohort, most patients (65%) were admitted to ICU post-operatively, 8% experienced post-operative delirium, 27% were discharged with an increase in care needs and 11% were readmitted to hospital within 30-days. However, there was no detectable significant difference in any of these outcomes between those with or without polypharmacy, nor those with or without a significant ACB score. Those with polypharmacy however, experienced a longer length of hospital stay (median 15 vs. 12 days).

In a multivariable logistic regression model adjusted for age, sex, Charlson Comorbidity Index score, frailty category and type of surgery, neither polypharmacy nor high anticholinergic burden appeared to be independently associated with one year mortality (Table 4). Polypharmacy had an adjusted odds ratio of 0.83 (95% confidence interval 0.28 to 2.33, *p* = 0.73) and high anticholinergic burden an adjusted odds ratio of 0.67 (95% confidence interval 0.13 to 2.80, *p* = 0.60).

**Table 4:** Multivariable logistic regression model

Predictor	Adjusted OR	95% CI	*p* value
Polypharmacy (≥ 5 medications)	0.83	0.28 to 2.33	0.726
High anticholinergic burden (ACB ≥ 3)	0.67	0.13 to 2.80	0.601
Age	1.05	0.98 to 1.12	0.206
Male sex	0.65	0.24 to 1.69	0.383
Charlson Comorbidity Index	1.15	0.89 to 1.50	0.279
Mild frailty	2.4	0.58 to 16.36	0.280
Moderate or severe frailty	4.6	0.92 to 34.87	0.086
Bowel resection	1.32	0.51 to 3.58	0.579

## Discussion

Emergency laparotomy carries a higher risk in older adults, and these patients require careful risk assessment before embarking upon surgery. A key component of risk stratification is consideration of multimorbidity which is prevalent in ageing populations and naturally associated with the prescription of multiple drugs [24]. Whilst there is no universal consensus on the definition of polypharmacy, the most used definition is the prescription of ≥ 5 drugs [25]. The prevalence of polypharmacy is estimated to be 23% in adults aged ≥ 65 years in the UK [26]. Polypharmacy has been previously associated with adverse patient outcomes including functional decline, hospitalisation due to fall and adverse drug events [4, 5, 27].

Polypharmacy exposes patients to a higher ACB, which is defined as the cumulative effect of taking medications with anticholinergic activity. Higher ACB arises with specific medications known to have high anticholinergic activity or with an accumulation of medications with low and medium anticholinergic burden. A high ACB has been associated with adverse outcomes such as cognitive decline and decline in physical function [6]. It could therefore be hypothesised that polypharmacy and elevated ACB lead to worsened outcomes in older adults undergoing emergency laparotomy.

Our study indicates that in the context of older patients undergoing emergency laparotomy, the presence of polypharmacy and high ACB score are common. Previous studies have shown varying prevalence rates. For example, in an elective surgical population, the prevalence of polypharmacy in older adults was 74% [28]. A Danish study of older adults estimated the 5-year incidence of polypharmacy to be 47% [29]. In our study, those with polypharmacy were characterised by older age, a higher proportion of moderate-to-severe frailty and a greater incidence of comorbidities including chronic respiratory disease, heart failure, stroke syndromes, diabetes mellitus and malignancy. This is likely reflective of polypharmacy being a parallel consequence of advancing age, frailty and comorbidity burden. In our study, neither polypharmacy nor significant ACB appeared to be associated with increased mortality observed at the various time points. Our findings mirror that of a previous observational study demonstrating that higher ACB was not associated with mortality up to 90 days [30]. A strength of our study is assessment of mortality up to one year, for which we found no detectable significant difference. Conversely, in a large retrospective cohort study of older adults, an increased mortality rate of 15% in patients with ARS score 1–2 and 14% in ARS score ≥ 3 has been observed [31].

Additionally, we showed no apparent increase in the incidence of Clavien Dindo I-IVb complications, post-operative delirium or ICU admission. A meta-analysis of three studies found polypharmacy, defined as ≥ 5 drugs, to be associated with 30-day postoperative complications defined as Clavien Dindo II or greater in older adults undergoing elective surgery for cancer patients [32]. Literature on the association of post-operative delirium is mixed. A randomised controlled intervention trial of 651 patients admitted for gastrointestinal, urogenital or gynaecological cancer surgery demonstrated that patients with significant ACB were 2.2 times more likely to develop post-operative delirium than those patients without [12]. On the contrary, a prospective observational study of 837 older adults undergoing a wide range of elective surgery found no correlation between ACB and development of post-operative delirium [33].

We demonstrated an increase in the median length of hospital stay in those with polypharmacy compared to those without (15 vs. 12 days, *p* = 0.025). This is an important outcome as additional days spent in hospital are associated with significant costs to health providers [34]. Furthermore, older adults are at inherent risk of nosocomial infections, delirium and physical decline with protracted hospitalisation [35]. A Japanese study of 584 patients undergoing gastrointestinal surgery found polypharmacy to be associated with increased length of hospital stay in adults aged over 50 [36]. Similar associations have been observed with ACB where in a study of 245,410 older adults undergoing elective non-cardiac surgery, stepwise increases in length of stay were associated with ACB, with mean length of stay of 7.8 days in ARS 1–2 versus 6.8 days for those patients with no ACB. In addition, cost of care and readmission rates also increased with ACB [31]. Whilst previous studies have also shown increases in readmission within 30-days, this was not observed in our study [30].

Most of the existing literature focuses on polypharmacy and ACB in a community or medical setting and data in surgical settings are limited. Although it may be intuitive that polypharmacy and associated ACB predict negative post-operative outcomes, definitive evidence is lacking for several reasons, not least the complexity of management of highly comorbid and frail patient groups who may already have other unfavourable characteristics predisposing them to adverse surgical outcomes. Another consideration is the NELA mandate that patients aged ≥ 65 years with important morbidity or frailty and all patients aged ≥ 80 years receive postoperative care by a consultant physician/geriatrician [37]. Comprehensive geriatric assessment (CGA) with medical and pharmacological intervention postoperatively is therefore becoming common practice in many institutions, including our own. Preliminary analysis of NELA data suggests that multidisciplinary management of this patient group can improve outcomes, with the greatest reduction in mortality rates over time observed in the oldest cohorts. This may be due to several interventions including increased perioperative medical input by experienced general physicians [16].

Previous studies have demonstrated that age and comorbidity burden are possible factors healthcare professionals consider when deciding if surgery is an appropriate intervention for an older adult [38–40]. As part of this, assessment of medication history may also naturally factor into this decision making. Our study has demonstrated that medication burden alone, as assessed by polypharmacy and/or ACB scoring, may not be a reason to deny emergency laparotomy in an older adult. These findings may be useful to clinicians in the context of using other validated risk stratification tools. We should also be mindful that emergency laparotomy is, by definition, physiologically urgent and lifesaving, with little room for preoperative optimisation, but plenty of scope for postoperative optimisation of morbidity, cognition, function and pharmacology.

There are limitations to our study. As a single-centre study, albeit at two geographically separate general hospitals within the same trust, we cannot generalise our results to other settings. Moreover, this research did not differentiate between appropriate versus inappropriate polypharmacy. Varying definitions of polypharmacy and ACB may affect comparability with other studies. The lower than anticipated incidence of delirium may be reflective of retrospective data collection and cognitive function not being formally assessed at regular intervals and/or consistently documented postoperatively. Additionally, the association between polypharmacy and increased length of hospital stay may be confounded by a greater proportion of those in the polypharmacy group having moderate-to-severe frailty. Considering the observational nature of our study a power analysis was not appropriate. Even though statistical significance was achieved for certain variables, the lack of significance in others does not necessarily mean that there was no association; instead, it may be attributed to the small sample size. Despite achieving significant results in several outcomes, we acknowledge the risk of a type 2 error occurring with our experimental sample size. Therefore, we were unable to discount an association between polypharmacy, ACB and those variables which did not achieve statistical significance with observed effect sizes.

## Conclusion

Polypharmacy and significant ACB are common in a heterogenous cohort of older patients undergoing emergency laparotomy. However, the presence of either of these did not appear to adversely affect outcomes apart from length of hospital stay. Confounding patient characteristics such as age, frailty and comorbidity burden, all predictors of physiological reserve, may collectively have greater impact on negative outcomes. Specific adverse outcomes associated with polypharmacy and ACB are therefore difficult to clarify in context. Polypharmacy and associated ACB may simply be considered as bystander, surrogate markers of vulnerability and poor prognosis. Practical interventions to optimise pharmacological management in older surgical patients are multidisciplinary team management with early and increased perioperative medical input by experienced general physicians with expertise in CGA and confidence in pharmacological rationalisation. Further well-designed studies should incorporate this phenomenon on a larger scale and investigate targeted interventions to improve outcomes in this often-overlooked population.

## Supplementary Information

Below is the link to the electronic supplementary material.

Supplementary File 1 (DOCX 14.1 KB)

Supplementary File 2 (DOCX 67.6 KB)

## Data Availability

No datasets were generated or analysed during the current study.
