# Supplementary material for: Polypharmacy and anticholinergic burden are common but not independently associated with outcomes after emergency laparotomy in older adults
Source: Langenbecks Arch Surg. 2026 Apr 21;411(1):150. doi: 10.1007/s00423-026-04060-z (PMC13230249; doi:10.1007/s00423-026-04060-z)
Supplement: Supplementary file 1 — (DOCX 14.1 KB) [file 423_2026_4060_MOESM1_ESM.docx]

**Supplementary File 1: NELA Inclusion/ Exclusion Criteria**

Patients with the following characteristics are included

- Aged 18 years and over,
- Who undergo an expedited, urgent or emergency (NCEPOD definitions) abdominal procedure on the gastrointestinal tract.

Including

- Open, laparoscopic, or laparoscopically-assisted procedures
- Procedures involving the stomach, small or large bowel, or rectum for conditions such as perforation, ischaemia, abdominal abscess, bleeding or obstruction
- Washout/evacuation of intra-peritoneal abscess (unless due to appendicitis or cholecystitis – excluded, see below)
- Washout/evacuation of intra-peritoneal haematoma
- Bowel resection/repair due to incarcerated incisional, umbilical, inguinal and femoral hernias (but not hernia repair without bowel resection/repair). E.g. Large incisional hernia repair with bowel resection
- Bowel resection/repair due to obstructing/incarcerated incisional hernias provided the presentation and findings were acute. This will include large incisional hernia repair with division of adhesions.
- Laparotomy/laparoscopy with inoperable pathology (e.g. peritoneal/hepatic metastases) where the intention was to perform a definitive procedure. This does not include purely diagnostic procedures.
- Laparoscopic/Open Adhesiolysis
- Return to theatre for repair of substantial dehiscence of major abdominal wound (i.e. “burst abdomen”)
- Return to theatre for complications that require the assistance of a general surgeon following either an interventional radiology procedure or following gynaecological oncology surgery.
- Any reoperation/return to theatre for complications of elective or non-elective general/upper GI surgery meeting the criteria above is included. Returns to theatre (apart from those interventional radiology or gynaecology-oncology complications described immediately above this point), for complications following non-GI surgery are excluded (see exclusion criteria below).

Patients with the following characteristics are excluded

- Patients under 18
- Elective laparotomy / laparoscopy
- Diagnostic laparotomy/laparoscopy where no subsequent procedure is performed (NB, if no procedure is performed because of inoperable pathology, then include)
- Appendicectomy +/- drainage of localised collection unless the procedure is incidental to a non-elective procedure on the GI tract
- Cholecystectomy +/- drainage of localised collection unless the procedure is incidental to a non-elective procedure on the GI tract
- Non-elective hernia repair without bowel resection or division of adhesions
- Minor abdominal wound dehiscence unless this causes bowel complications requiring resection
- Non-elective formation of a colostomy or ileostomy as either a trephine or a laparoscopic procedure
