# Supplementary material for: Polypharmacy and anticholinergic burden are common but not independently associated with outcomes after emergency laparotomy in older adults
Source: Langenbecks Arch Surg. 2026 Apr 21;411(1):150. doi: 10.1007/s00423-026-04060-z (PMC13230249; doi:10.1007/s00423-026-04060-z)
Supplement: Supplementary file 2 — (DOCX 67.6 KB) [file 423_2026_4060_MOESM2_ESM.docx]

**Supplementary File 2: Anticholinergic burden scores of medications**

| **Name** | **Score** |
| --- | --- |
| 5-hydroxytryptophan | 0 |
| Abacavir | 0 |
| Abiraterone | 0 |
| Acamprosate | 0 |
| Acarbose | 0 |
| Acebutolol | 0 |
| Acetaminophen | 0 |
| Acetazolamide | 0 |
| Acetic acid | 0 |
| Acetohexamide | 0 |
| Acetylcarnitine | 0 |
| Acetylcysteine | 0 |
| Aciclovir | 0 |
| Acidophilus | 0 |
| Acitretin | 0 |
| Aclidinium | 0 |
| Acyclovir | 0 |
| Acyl carnitine | 0 |
| Adalimumab | 0 |
| Adefovir dipivoxil | 0 |
| Adenosine | 0 |
| Adrenaline | 0 |
| Agomelatine | 0 |
| Al hydroxide/mg hydroxide/simethicone | 0 |
| Albumin human | 0 |
| Albuterol | 0 |
| Alclometasone topical | 0 |
| Alendronate | 0 |
| Alfuzosin | 0 |
| Alginic acid | 0 |
| Alimemazine | 1 |
| Aliskiren | 0 |
| Allopurinol | 0 |
| Aloe vera | 0 |
| Alpha-d-galactosidase | 0 |
| Alprazolam | 1 |
| Alprostadil | 0 |
| Alteplase | 0 |
| Aluminum acetate topical | 0 |
| Aluminum carbonate | 0 |
| Aluminum hydroxide | 0 |
| Alverine | 1 |
| Alzelaic acid | 0 |
| Amantadine | 1 |
| Amiloride | 0 |
| Amino acids | 0 |
| Aminophylline | 1 |
| Amiodarone | 0 |
| Amitriptyline | 3 |
| Amlodipine | 0 |
| Ammonium lactate topical | 0 |
| Amoxapine | 3 |
| Amoxicillin | 0 |
| Amoxicillin-clavulanate | 0 |
| Amphetamine | 0 |
| Amphotericin b | 0 |
| Ampicillin | 1 |
| Anagrelide | 0 |
| Anakinra | 0 |
| Anastrozole | 0 |
| Apap/dichloralphenazone/isometheptene | 0 |
| Apixaban | 0 |
| Apraclonidine ophthalmic | 0 |
| Apremilast | 0 |
| Arginine | 0 |
| Aripiprazole | 1 |
| Ascorbic acid | 0 |
| Asenapine | 1 |
| Aspirin | 0 |
| Atazanavir | 0 |
| Atenolol | 0 |
| Atomoxetine | 0 |
| Atorvastatin | 0 |
| Atropine | 3 |
| Attapulgite | 0 |
| Azatadine | 3 |
| Azathioprine | 0 |
| Azelastine nasal | 1 |
| Azelastine ophthalmic | 1 |
| Azilsartan medoxomil | 0 |
| Azithromycin | 0 |
| Bacitracin | 0 |
| Baclofen | 0 |
| Balsalazide | 0 |
| Balsam peru topical | 0 |
| Barberry | 1 |
| Becaplermin topical | 0 |
| Beclomethasone | 0 |
| Bee pollen | 0 |
| Belladonna | 3 |
| Benzocaine topical | 0 |
| Benzocaine/butamben/tetracaine topical | 0 |
| Benzonatate | 0 |
| Benztropine | 3 |
| Beta-carotene | 0 |
| Betahistine | 0 |
| Betaine | 0 |
| Betamethasone | 0 |
| Betamethasone topical | 0 |
| Betamethasone-clotrimazole topical | 0 |
| Betaxolol | 0 |
| Bethanechol | 0 |
| Bevacizumab | 0 |
| Bicalutamide | 0 |
| Bilastine | 0 |
| Bilberry | 0 |
| Bimatoprost ophthalmic | 0 |
| Bioflavonoids | 0 |
| Biotin | 0 |
| Biperiden | 3 |
| Bisacodyl | 0 |
| Bismuth subsalicylate | 0 |
| Bisoprolol | 0 |
| Black cohosh | 0 |
| Bosentan | 0 |
| Brexpiprazole | 0 |
| Brimonidine ophthalmic | 0 |
| Brinzolamide ophthalmic | 0 |
| Bromazepam | 0 |
| Bromhexine | 0 |
| Bromocriptine | 1 |
| Brompheniramine | 3 |
| Buclizine | 3 |
| Budesonide | 0 |
| Bumetanide | 0 |
| Bupivacaine | 0 |
| Buprenorphine | 0 |
| Bupropion | 1 |
| Buspirone | 0 |
| Butabarbital | 0 |
| Butalbital | 0 |
| Butenafine | 0 |
| Butoconazole topical | 0 |
| Butorphanol | 0 |
| Butylated hydroxytoluene topical | 0 |
| Cabergoline | 0 |
| Cadexomer iodine | 0 |
| Caffeine | 0 |
| Calamine topical | 0 |
| Calcipotriene topical | 0 |
| Calcitonin | 0 |
| Calcitriol | 0 |
| Calcium acetate | 0 |
| Calcium carbonate | 0 |
| Calcium-vitamin d | 0 |
| Camphor topical | 0 |
| Camphor-menthol topical | 0 |
| Canagliflozin | 0 |
| Candesartan | 0 |
| Cannabidiol | 0 |
| Capecitabine | 0 |
| Capsaicin topical | 0 |
| Captopril | 1 |
| Carbachol ophthalmic | 0 |
| Carbamazepine | 0 |
| Carbamide peroxide otic | 0 |
| Carbidopa | 0 |
| Carbidopa-levodopa | 1 |
| Carbimazole | 0 |
| Carbinoxamine | 3 |
| Carboplatin | 0 |
| Carisoprodol | 0 |
| Carmellose | 0 |
| Carnitine | 0 |
| Carvedilol | 0 |
| Casanthranol | 0 |
| Casanthranol-docusate | 0 |
| Cascara sagrada | 0 |
| Caspofungin | 0 |
| Castor oil | 0 |
| Cat's claw | 0 |
| Cefaclor | 0 |
| Cefadroxil | 0 |
| Cefamandole | 1 |
| Cefazolin | 0 |
| Cefepime | 0 |
| Cefixime | 0 |
| Cefotetan | 0 |
| Cefoxitin | 1 |
| Ceftibuten | 0 |
| Ceftriaxone | 0 |
| Cefuroxime | 0 |
| Celecoxib | 1 |
| Cephalexin | 0 |
| Cephalothin | 1 |
| Cerivastatin | 0 |
| Cetirizine | 1 |
| Cetylpyridinium topical | 0 |
| Cevimeline | 0 |
| Chamomile | 0 |
| Charcoal | 0 |
| Charcoal-sorbitol | 0 |
| Chloral hydrate | 0 |
| Chlorambucil | 0 |
| Chloramphenicol | 0 |
| Chlordiazepoxide | 1 |
| Chlorhexidine topical | 0 |
| Chloroquine | 1 |
| Chlorothiazide | 0 |
| Chlorphenamine | 3 |
| Chlorpromazine | 3 |
| Chlorpropamide | 0 |
| Chlorprothixene | 3 |
| Chlortalidone | 1 |
| Chlorzoxazone | 0 |
| Cholecalciferol | 0 |
| Cholestyramine | 0 |
| Choline salicylate | 0 |
| Chondroitin | 0 |
| Chromium picolinate | 0 |
| Ciclesonide | 0 |
| Ciclopirox topical | 0 |
| Ciclosporin | 1 |
| Cilastatin | 0 |
| Cilexetil | 0 |
| Cilostazol | 0 |
| Cimetidine | 1 |
| Cinacalcet | 0 |
| Cinnarizine | 3 |
| Ciprofloxacin | 0 |
| Cisapride | 0 |
| Cisplatin | 0 |
| Citalopram | 1 |
| Citric acid | 0 |
| Clarithromycin | 0 |
| Clavulanate | 0 |
| Clemastine | 3 |
| Clidinium | 3 |
| Clindamycin | 1 |
| Clindamycin cream or gel | 0 |
| Clobazam | 0 |
| Clobetasol topical | 0 |
| Clodronate | 0 |
| Clofazimine | 0 |
| Clomipramine | 3 |
| Clonazepam | 1 |
| Clonidine | 0 |
| Clopidogrel | 0 |
| Clorazepate | 1 |
| Clotrimazole | 0 |
| Cloxacillin | 0 |
| Clozapine | 3 |
| Co-codamol | 0 |
| Coal tar topical | 0 |
| Cod liver oil | 0 |
| Codeine | 0 |
| Coenzyme q10 | 0 |
| Colchicine | 0 |
| Colecalciferol | 0 |
| Colesevelam | 0 |
| Colestipol | 0 |
| Colistin sulfate otic | 0 |
| Collagenase topical | 0 |
| Conjugated estrogens | 0 |
| Conjugated estrogens topical | 0 |
| Corticosterone | 1 |
| Cortisone | 1 |
| Cosyntropin | 0 |
| Cranberry | 0 |
| Cromolyn | 0 |
| Curcumin | 0 |
| Cyanocobalamin | 0 |
| Cyclizine | 3 |
| Cyclobenzaprine | 3 |
| Cyclophosphamide | 0 |
| Cycloserine | 1 |
| Cyclosporin eye drops | 0 |
| Cyclosporine | 1 |
| Cyproheptadine | 3 |
| Cyproterone acetate | 0 |
| Dabigatran | 0 |
| Dalteparin | 0 |
| Danazol | 0 |
| Dandelion | 0 |
| Dantrolene | 0 |
| Dapagiflozin | 0 |
| Dapsone | 0 |
| Daptomycin | 0 |
| Darbepoetin alfa | 0 |
| Darifenacin | 3 |
| Darunavir | 0 |
| Dehydroepiandrosterone | 0 |
| Demeclocycline | 0 |
| Denosumab | 0 |
| Desipramine | 3 |
| Desloratadine | 1 |
| Desmopressin | 0 |
| Desonide topical | 0 |
| Desoximetasone topical | 0 |
| Desvenlafaxine | 1 |
| Dexamethasone | 1 |
| Dexamethasone nasal | 0 |
| Dexamethasone ophthalmic | 0 |
| Dexamethasone topical | 0 |
| Dexbrompheniramine | 3 |
| Dexchlorpheniramine | 3 |
| Dexlansoprazole | 0 |
| Dextroamphetamine | 0 |
| Dextromethorphan | 1 |
| Diazepam | 1 |
| Dibucaine topical | 0 |
| Diclofenac | 0 |
| Diclofenac-misoprostol | 0 |
| Dicloxacillin | 0 |
| Dicycloverine | 3 |
| Didanosine | 0 |
| Dienestrol topical | 0 |
| Diflunisal | 0 |
| Digitoxin | 1 |
| Digoxin | 1 |
| Dihydroxyaluminum sodium carbonate | 0 |
| Diltiazem | 0 |
| Dimenhydrinate | 3 |
| Dimeticone | 0 |
| Dimetindene | 1 |
| Diphenhydramine | 3 |
| Diphenhydramine cream | 1 |
| Diphenoxylate | 0 |
| Dipivefrin ophthalmic | 0 |
| Dipyridamole | 0 |
| Dirithromycin | 0 |
| Disopyramide | 2 |
| Disulfiram | 0 |
| Divalproex sodium | 0 |
| Dobutamine | 0 |
| Docosanol topical | 0 |
| Docusate | 0 |
| Docusate-senna | 0 |
| Dofetilide | 0 |
| Domperidone | 0 |
| Donepezil | 0 |
| Dopamine | 0 |
| Dorzolamide ophthalmic | 0 |
| Dosulepin | 3 |
| Doxazosin | 0 |
| Doxepin | 3 |
| Doxercalciferol | 0 |
| Doxycycline | 0 |
| Doxylamine | 3 |
| Dronedarone | 0 |
| Dulaglutide | 0 |
| Duloxetine | 0 |
| Dutaseride | 0 |
| Dydrogesterone | 0 |
| Dyphylline | 0 |
| Echinacea | 0 |
| Econazole topical | 0 |
| Edoxaban | 0 |
| Edrophonium | 0 |
| Efavirenz | 0 |
| Efinaconazole topical | 0 |
| Eflornithine topical | 0 |
| Eletriptan | 0 |
| Emollients | 0 |
| Empagliflozin | 0 |
| Emtricitabine | 0 |
| Enalapril | 0 |
| Enoxaparin | 0 |
| Enriched soy lecithin | 0 |
| Entacapone | 1 |
| Ephedrine | 0 |
| Epinastine ophthalmic | 0 |
| Epinephrine | 0 |
| Eplerenone | 0 |
| Epoetin alfa | 0 |
| Eprosartan | 0 |
| Ergocalciferol | 0 |
| Ergoloid mesylates | 0 |
| Ergotamine | 1 |
| Erlotinib | 0 |
| Ertapenem | 0 |
| Erythromycin | 0 |
| Escitalopram | 1 |
| Esomeprazole | 0 |
| Estazolam | 0 |
| Esterified estrogens | 0 |
| Estradiol | 0 |
| Estradiol topical | 0 |
| Estriol | 0 |
| Estropipate | 0 |
| Eszopiclone | 0 |
| Etanercept | 0 |
| Ethambutol | 0 |
| Ethinyl estradiol | 0 |
| Etidronate | 0 |
| Etodolac | 0 |
| Etomidate | 0 |
| Etoposide | 0 |
| Etoricoxib | 1 |
| Evening primrose | 0 |
| Evolocumab | 0 |
| Exemestane | 0 |
| Exenatide | 0 |
| Ezetimibe | 0 |
| Factor ix complex | 0 |
| Famciclovir | 0 |
| Famotidine | 0 |
| Febuxostat | 0 |
| Felbamate | 0 |
| Felodipine | 0 |
| Fenofibrate | 0 |
| Fenoprofen | 0 |
| Fentanyl | 1 |
| Fentanyl topical | 1 |
| Ferrous fumarate | 0 |
| Ferrous gluconate | 0 |
| Ferrous sulfate | 0 |
| Fesoterodine | 3 |
| Fexofenadine | 0 |
| Filgrastim | 0 |
| Finasteride | 0 |
| Flavoxate | 3 |
| Flax | 0 |
| Flecainide | 0 |
| Flucloxacillin | 0 |
| Fluconazole | 0 |
| Fludrocortisone | 0 |
| Flumazenil | 0 |
| Flunisolide | 0 |
| Flunitrazepam | 1 |
| Flunizepam | 0 |
| Fluocinolone topical | 0 |
| Fluocinonide topical | 0 |
| Fluoride | 0 |
| Fluoride topical | 0 |
| Fluorometholone ophthalmic | 0 |
| Fluorouracil | 0 |
| Fluoxetine | 1 |
| Fluphenazine | 1 |
| Flurazepam | 1 |
| Flurbiprofen | 0 |
| Flutamide | 0 |
| Fluticasone | 0 |
| Fluticasone-salmeterol | 0 |
| Fluvastatin | 0 |
| Fluvoxamine | 1 |
| Folic acid | 0 |
| Formoterol | 0 |
| Fosinopril | 0 |
| Fosphenytoin | 0 |
| Framycetin sulphate | 0 |
| Furosemide | 0 |
| Gabapentin | 0 |
| Galantamine | 0 |
| Ganciclovir | 0 |
| Garlic | 0 |
| Gatifloxacin | 0 |
| Gemfibrozil | 0 |
| Gemifloxacin | 0 |
| Gentamicin | 1 |
| Gentamicin ophthalmic | 0 |
| Gentamicin topical | 0 |
| Ginger | 0 |
| Ginkgo | 0 |
| Ginseng | 0 |
| Glatiramer | 0 |
| Glibenclamide | 0 |
| Gliclazide | 0 |
| Glimepiride | 0 |
| Glipizide | 0 |
| Glucagon | 0 |
| Glucosamine | 0 |
| Glucose | 0 |
| Glutamic acid | 0 |
| Glyburide | 0 |
| Glyceril trinitrate | 0 |
| Glycerin | 0 |
| Glycine | 0 |
| Glycopyrronium - inhaled | 1 |
| Glycopyrronium injectable | 3 |
| Goserelin | 0 |
| Gotu kola | 0 |
| Gramicidin ophthalmic | 0 |
| Grape seed oil | 0 |
| Green tea | 0 |
| Griseofulvin | 0 |
| Guaifenesin | 1 |
| Guanabenz | 0 |
| Guanfacine | 0 |
| Halcinonide topical | 0 |
| Halobetasol topical | 0 |
| Haloperidol | 1 |
| Hawthorn | 0 |
| Heme iron polypeptide | 0 |
| Heparin | 0 |
| Hetastarch | 0 |
| Hexachlorophene topical | 0 |
| Holy basil | 0 |
| Homatropine | 3 |
| Homatropine ophthalmic | 2 |
| Horse chestnut | 0 |
| Huperzine | 0 |
| Hydralazine | 1 |
| Hydrochlorothiazide | 0 |
| Hydrocodone | 0 |
| Hydrocortisone | 1 |
| Hydrocortisone ophthalmic | 0 |
| Hydrocortisone otic | 0 |
| Hydrocortisone topical | 0 |
| Hydrogen peroxide topical | 0 |
| Hydromorphone | 0 |
| Hydroxychloroquine | 0 |
| Hydroxypropyl methylcellulose ophthalmic | 0 |
| Hydroxyquinoline topical | 0 |
| Hydroxyurea | 0 |
| Hydroxyzine | 1 |
| Hyoscine butylbromide | 3 |
| Hyoscine hydrobromide | 3 |
| Hyoscyamine | 3 |
| Hypromellose | 0 |
| Ibandronate | 0 |
| Ibuprofen | 0 |
| Iloperidone | 1 |
| Imatinib | 0 |
| Imipenem | 0 |
| Imipenem-cilastatin | 0 |
| Imipramine | 3 |
| Imiquimod topical | 0 |
| Immune globulin intramuscular | 0 |
| Immune globulin intravenous | 0 |
| Immune globulin subcutaneous | 0 |
| Indacaterol | 0 |
| Indapamide | 0 |
| Indomethacin | 0 |
| Infliximab | 0 |
| Influenza virus vaccine | 0 |
| Inosine | 0 |
| Inositol | 0 |
| Insulin | 0 |
| Interferon beta-1a | 0 |
| Interferon beta-1b | 0 |
| Inuflora | 0 |
| Ipratropium | 1 |
| Ipratropium nasal | 1 |
| Irbesartan | 0 |
| Irinotecan | 0 |
| Iron polysaccharide | 0 |
| Isoniazid | 0 |
| Isopropyl alcohol topical | 0 |
| Isosorbide | 1 |
| Isosorbide dinitrate | 1 |
| Isosorbide mononitrate | 1 |
| Isotretinoin | 0 |
| Isradipine | 0 |
| Itraconazole | 0 |
| Kaolin | 0 |
| Ketamine | 3 |
| Ketoconazole | 0 |
| Ketoprofen | 0 |
| Ketoralac | 0 |
| Ketorolac ophthalmic | 0 |
| Ketotifen ophthalmic | 1 |
| L-histadine | 0 |
| L-theanine | 0 |
| L-tyrosine | 0 |
| Labetalol | 0 |
| Lactase | 0 |
| Lactic acid topical | 0 |
| Lactobacillus acidophilus | 0 |
| Lactulose | 0 |
| Lamivudine | 0 |
| Lamotrigine | 0 |
| Lanolin-mineral oil topical | 0 |
| Lansoprazole | 1 |
| Lanthanum carbonate | 0 |
| Latanoprost ophthalmic | 0 |
| Lecithin | 0 |
| Leflunomide | 0 |
| Lercanidipine | 0 |
| Letrozole | 0 |
| Leucovorin | 0 |
| Leuprolide | 0 |
| Levalbuterol | 0 |
| Levetiracetam | 0 |
| Levmetamfetamine nasal | 0 |
| Levobunolol ophthalmic | 0 |
| Levocarnitine | 0 |
| Levocetirizine | 1 |
| Levodopa | 1 |
| Levofloxacin | 0 |
| Levomepromazine | 3 |
| Levothyroxine | 0 |
| Licorice | 0 |
| Lidocaine | 0 |
| Linaclotide | 0 |
| Linagliptin | 0 |
| Lindane topical | 0 |
| Linezolid | 0 |
| Liothyronine | 0 |
| Liotrix | 0 |
| Lipoic acid | 0 |
| Liraglutide | 0 |
| Lisinopril | 0 |
| Lithium | 1 |
| Lodoxamide ophthalmic | 0 |
| Loperamide | 1 |
| Loracarbef | 0 |
| Loratadine | 1 |
| Lorazepam | 1 |
| Losartan | 0 |
| Loteprednol ophthalmic | 0 |
| Lovastatin | 0 |
| Loxapine | 2 |
| Lubiprostone | 0 |
| Lutein | 0 |
| Lvp solution | 0 |
| Lycopene | 0 |
| Lysine | 0 |
| Magnesium amino acids chelate | 0 |
| Magnesium carbonate | 0 |
| Magnesium chloride | 0 |
| Magnesium citrate | 0 |
| Magnesium gluconate | 0 |
| Magnesium glycinate | 0 |
| Magnesium hydroxide | 0 |
| Magnesium oxide | 0 |
| Magnesium salicylate | 0 |
| Magnesium sulfate | 0 |
| Magnesium trisilicate | 0 |
| Manganese | 0 |
| Mannitol | 0 |
| Maprotiline | 2 |
| Mebeverine | 0 |
| Meclizine | 1 |
| Medroxyprogesterone | 0 |
| Mefloquine | 0 |
| Megestrol | 0 |
| Melatonin | 0 |
| Meloxicam | 0 |
| Melphalan | 0 |
| Memantine | 0 |
| Menthol topical | 0 |
| Meperidine | 0 |
| Meprobamate | 0 |
| Mequinol | 0 |
| Mercaptopurine | 0 |
| Meropenem | 0 |
| Mesalazine | 0 |
| Mesoridazine | 2 |
| Mestranol | 0 |
| Metaproterenol | 0 |
| Metaxalone | 0 |
| Metformin | 1 |
| Methadone | 1 |
| Methazolamide | 0 |
| Methenamine | 0 |
| Methenamine-sodium biphosphate | 0 |
| Methimazole | 0 |
| Methocarbamol | 3 |
| Methotrexate | 0 |
| Methotrimeprazine | 2 |
| Methscopolamine | 3 |
| Methyclothiazide | 0 |
| Methyl salicylate topical | 0 |
| Methylcellulose | 0 |
| Methyldopa | 0 |
| Methylene blue | 0 |
| Methylphenidate | 0 |
| Methylprednisolone | 1 |
| Methylprednisolone topical | 0 |
| Methylsulfonylmethane | 0 |
| Methyltestostone | 0 |
| Metipranolol ophthalmic | 0 |
| Metoclopramide | 0 |
| Metolazone | 0 |
| Metoprolol | 0 |
| Metronidazole | 0 |
| Mexiletine | 0 |
| Mianserin | 0 |
| Miconazole | 0 |
| Midazolam | 1 |
| Midodrine | 0 |
| Miglitol | 0 |
| Milk thistle | 0 |
| Mineral oil | 0 |
| Minocycline | 0 |
| Minoxidil | 0 |
| Mirabegron | 0 |
| Mirtazapine | 1 |
| Misoprostol | 0 |
| Modafinil | 0 |
| Moexipril | 0 |
| Molindone | 0 |
| Mometasone | 0 |
| Montelukast | 0 |
| Moricizine | 0 |
| Morphine | 1 |
| Moxifloxacin | 0 |
| Moxonidine | 0 |
| Multivitamin | 0 |
| Mupirocin topical | 0 |
| Mycophenolate mofetil | 0 |
| Mycophenolic acid | 0 |
| N-acetyl-tyrosine | 0 |
| Nabilone | 0 |
| Nabumetone | 0 |
| Nadolol | 0 |
| Nalbuphine | 1 |
| Naloxone | 0 |
| Naltrexone | 0 |
| Naphazoline ophthalmic | 0 |
| Naproxen | 0 |
| Naratriptan | 1 |
| Nateglinide | 0 |
| Nebivolol | 0 |
| Nefazodone | 0 |
| Nefopam | 2 |
| Neomycin | 1 |
| Neomycin ophthalmic | 0 |
| Neomycin topical | 0 |
| Neostigmine | 0 |
| Nettles | 0 |
| Nevirapine | 0 |
| Niacin | 0 |
| Nicardipine | 0 |
| Nicotinamide riboside | 0 |
| Nicotine | 0 |
| Nifedipine | 0 |
| Nimodipine | 0 |
| Nisoldipine | 0 |
| Nitrazepam | 1 |
| Nitrofurantoin | 0 |
| Nitroglycerin | 0 |
| Nitroprusside | 0 |
| Nizatidine | 1 |
| Norepinephrine | 0 |
| Norethisterone | 0 |
| Norfloxacin | 0 |
| Nortriptyline | 3 |
| Nystatin | 0 |
| Octreotide | 0 |
| Ocular lubricant | 0 |
| Ofloxacin | 0 |
| Olanzapine | 3 |
| Olmesartan | 0 |
| Olodaterol | 0 |
| Olopatadine ophthalmic | 1 |
| Omega-3 polyunsaturated fatty acids | 0 |
| Omega-3-ethyl esters | 0 |
| Omeprazole | 1 |
| Ondansetron | 0 |
| Opipramol | 2 |
| Orlistat | 0 |
| Orphenadrine | 3 |
| Orthosilicic acid | 0 |
| Oseltamivir | 0 |
| Oxaprozin | 0 |
| Oxazepam | 1 |
| Oxcarbazepine | 0 |
| Oxiconazole topical | 0 |
| Oxybutynin | 3 |
| Oxycodone | 1 |
| Oxymetazoline nasal | 0 |
| Paliperidone | 0 |
| Pamabrom | 0 |
| Pamidronate | 0 |
| Pancrelipase | 0 |
| Pancuronium | 1 |
| Pantoprazole | 1 |
| Papain-urea topical | 0 |
| Papaverine | 0 |
| Papaya | 0 |
| Paracetamol | 0 |
| Paricalcitol | 0 |
| Paroxetine | 3 |
| Pectin | 0 |
| Penicillin | 0 |
| Pentazocine | 0 |
| Pentoxifylline | 0 |
| Pergolide | 0 |
| Pericyazine | 3 |
| Perindopril | 0 |
| Permethrin topical | 0 |
| Perphenazine | 2 |
| Pethidine | 2 |
| Petrolatum topical | 0 |
| Phenazopyridine | 0 |
| Phenelzine | 1 |
| Phenindamine | 3 |
| Pheniramine | 3 |
| Pheniramine ophthalmic | 1 |
| Phenobarbitol | 1 |
| Phenol topical | 0 |
| Phenolphthalein | 0 |
| Phentermine | 0 |
| Phentolamine | 0 |
| Phenyl salicylate | 0 |
| Phenylephrine | 0 |
| Phenylpropanolamine | 0 |
| Phenyltoloxamine | 3 |
| Phenytoin | 0 |
| Phosphatidyl serine | 0 |
| Phosphorated carbohydrate solution | 0 |
| Phytonadione | 0 |
| Pilocarpine | 0 |
| Pimecrolimus topical | 0 |
| Pimozide | 2 |
| Pindolol | 0 |
| Pioglitazone | 0 |
| Piperacillin | 1 |
| Pirbuterol | 0 |
| Piroxicam | 0 |
| Pivampicillin | 0 |
| Podophyllum | 0 |
| Polycarbophil | 0 |
| Polyethylene glycol 3350 | 0 |
| Polyethylene glycol 3350 with electrolytes | 0 |
| Polymyxin b ophthalmic | 0 |
| Polymyxin b topical | 0 |
| Potassium acetate | 0 |
| Potassium bicarbonate | 0 |
| Potassium bicarbonate-potassium citrate | 0 |
| Potassium chloride | 0 |
| Potassium citrate | 0 |
| Potassium gluconate | 0 |
| Potassium iodide | 0 |
| Potassium phosphate | 0 |
| Potassium phosphate-sodium phosphate | 0 |
| Pramipexole | 1 |
| Pramlintide | 0 |
| Pramoxine topical | 0 |
| Pramoxine-zinc oxide topical | 0 |
| Pravastatin | 0 |
| Prazosin | 0 |
| Prednisolone | 1 |
| Prednisolone eye drops | 0 |
| Pregabalin | 0 |
| Pregnenolone | 0 |
| Prilocaine topical | 0 |
| Primidone | 0 |
| Probenecid | 0 |
| Probiotic | 0 |
| Procainamide | 1 |
| Prochlorperazine | 1 |
| Procyclidine | 3 |
| Progesterone | 0 |
| Promethazine | 3 |
| Propafenone | 0 |
| Propantheline | 3 |
| Propiverine | 3 |
| Propofol | 0 |
| Propoxyphene | 0 |
| Propranolol | 0 |
| Propylene glycol | 0 |
| Propylthiouracil | 0 |
| Protamine | 0 |
| Protriptyline | 3 |
| Pseudoephedrine | 0 |
| Psyllium | 0 |
| Pycnogenol | 0 |
| Pygeum | 0 |
| Pyrazinamide | 0 |
| Pyridostigmine | 0 |
| Pyridoxine | 0 |
| Pyrilamine | 3 |
| Quetiapine | 3 |
| Quinapril | 0 |
| Quinidine | 1 |
| Quinine | 0 |
| Quinine phenylethylbarbiturate | 0 |
| Rabeprazole | 0 |
| Raloxifene | 0 |
| Ramelteon | 0 |
| Ramipril | 0 |
| Ranitidine | 1 |
| Rasagiline | 0 |
| Reboxetine | 0 |
| Red yeast rice | 0 |
| Repaglinide | 0 |
| Reserpine | 0 |
| Resveratrol | 0 |
| Rhodiola rosea | 0 |
| Ribavirin | 0 |
| Rifampin | 0 |
| Rilpivirine | 0 |
| Rimantadine | 0 |
| Rimexolone ophthalmic | 0 |
| Risedronate | 0 |
| Risperidone | 1 |
| Ritonavir | 0 |
| Rivaroxaban | 0 |
| Rivastigmine | 0 |
| Rizatriptan | 0 |
| Rofecoxib | 0 |
| Ropinirole | 0 |
| Rosiglitazone | 0 |
| Rosuvastatin | 0 |
| Rotigotine | 1 |
| Roxythromycin | 0 |
| Royal jelly | 0 |
| S-adenosylmethionine | 0 |
| Salbutamol | 0 |
| Salicylamide | 0 |
| Salicylic acid topical | 0 |
| Salmeterol | 0 |
| Salsalate | 0 |
| Sargramostim | 0 |
| Sarilumab | 0 |
| Saw palmetto | 0 |
| Saxagliptin | 0 |
| Scopolamine | 3 |
| Scopolamine topical | 3 |
| Selegiline | 1 |
| Selenium | 0 |
| Senna | 0 |
| Sertraline | 1 |
| Sevelamer | 0 |
| Shark cartilage | 0 |
| Sibutramine | 0 |
| Sildenafil | 0 |
| Silodosin | 0 |
| Silver sulfadiazine topical | 0 |
| Simethicone | 0 |
| Simvastatin | 0 |
| Sirolimus | 0 |
| Sitagliptin | 0 |
| Sodium bicarbonate | 0 |
| Sodium biphosphate-sodium phosphate | 0 |
| Sodium chloride | 0 |
| Sodium chloride nasal | 0 |
| Sodium chloride, hypertonic, ophthalmic | 0 |
| Sodium citrate | 0 |
| Sodium ferric gluconate complex | 0 |
| Sodium hyaluronate | 0 |
| Sodium phosphate | 0 |
| Sodium picosulfate | 0 |
| Sodium polystyrene sulfonate | 0 |
| Sofosbuvir | 0 |
| Solifenacin | 3 |
| Sorbitol | 0 |
| Sotalol | 0 |
| Spironolactone | 0 |
| St. john's wort | 0 |
| Stavudine | 0 |
| Strontium | 0 |
| Succinylcholine | 0 |
| Sucralfate | 0 |
| Sulfacetamide sodium ophthalmic | 0 |
| Sulfacetamide sodium topical | 0 |
| Sulfadiazine | 0 |
| Sulfamethizole | 0 |
| Sulfamethoxazole | 0 |
| Sulfamethoxazole-trimethoprim | 0 |
| Sulfasalazine | 0 |
| Sulfur topical | 0 |
| Sulindac | 0 |
| Sumatriptan | 1 |
| Tacrine | 0 |
| Tacrolimus | 0 |
| Tadalafil | 0 |
| Tamoxifen | 0 |
| Tamsulosin | 0 |
| Taurine | 0 |
| Tazarotene topical | 0 |
| Tazobactam | 0 |
| Tegaserod | 0 |
| Telithromycin | 0 |
| Telmisartan | 0 |
| Temazepam. | 1 |
| Temozolomide | 0 |
| Tenofovir | 0 |
| Terazosin | 0 |
| Terbinafine | 0 |
| Terbutaline | 0 |
| Terconazole topical | 0 |
| Teriparatide | 0 |
| Testosterone | 0 |
| Tetanus-diphtheria toxoids | 0 |
| Tetracycline | 0 |
| Tetrahydrozoline ophthalmic | 0 |
| Thalidomide | 0 |
| Theophylline | 1 |
| Thiamazole | 0 |
| Thiamine | 0 |
| Thioridazine | 3 |
| Thiothixene | 0 |
| Thonzonium bromide otic | 0 |
| Thyrotropin alpha | 0 |
| Thyroxine | 0 |
| Tiagabine | 1 |
| Tiaprofenic acid | 0 |
| Tibolone | 0 |
| Ticarcillin | 0 |
| Ticlopidine | 0 |
| Timolol | 0 |
| Tiotropium | 1 |
| Tizanidine | 0 |
| Tobramycin | 1 |
| Tobramycin ophthalmic | 0 |
| Tofacitinib | 0 |
| Tolazamide | 0 |
| Tolbutamide | 0 |
| Tolcapone | 0 |
| Tolmetin | 0 |
| Tolterodine | 3 |
| Topiramate | 0 |
| Torsemide | 0 |
| Tramadol | 2 |
| Trandolapril | 1 |
| Tranexamic acid | 0 |
| Tranylcypromine | 1 |
| Travoprost ophthalmic | 0 |
| Trazodone | 0 |
| Tretinoin topical | 0 |
| Triamcinolone | 1 |
| Triamcinolone nasal | 0 |
| Triamcinolone topical | 0 |
| Triamterene | 1 |
| Triazolam | 1 |
| Tribulus terrestris | 0 |
| Trichlormethiazide | 0 |
| Triethanolamine polypeptide oleate otic | 0 |
| Trifluoperazine | 2 |
| Trifluperidol | 0 |
| Triflupromazine | 3 |
| Trihexyphenidyl | 3 |
| Trimethobenzamide | 3 |
| Trimethoprim | 0 |
| Trimipramine | 3 |
| Triprolidine | 3 |
| Troglitazone | 0 |
| Trolamine salicylate topical | 0 |
| Tropisetron | 0 |
| Trospium | 3 |
| Trypsin | 0 |
| Trypsin topical | 0 |
| Tuberculin purified protein derivative | 0 |
| Turmeric | 0 |
| Ubiquinone | 0 |
| Umeclidinium | 1 |
| Unoprostone ophthalmic | 0 |
| Urea topical | 0 |
| Ursodiol | 0 |
| Valacyclovir | 0 |
| Valdecoxib | 0 |
| Valerian | 0 |
| Valproic acid | 1 |
| Valsartan | 0 |
| Vancomycin | 1 |
| Vardenafil | 0 |
| Varenicline | 0 |
| Vasopressin | 0 |
| Vecuronium | 0 |
| Velpatasvir | 0 |
| Vemurafenib | 0 |
| Venlafaxine | 1 |
| Verapamil | 0 |
| Vigabatrin | 0 |
| Vilanterol | 0 |
| Vilazodone | 0 |
| Vincristine | 0 |
| Vinpocetine | 0 |
| Vitamin a | 0 |
| Vitamin a & d topical | 0 |
| Vitamin e | 0 |
| Voriconazole | 0 |
| Vortioxetine | 0 |
| Warfarin | 0 |
| Wheat dextrin | 0 |
| Wild yam | 0 |
| Witch hazel topical | 0 |
| Yohimbine | 0 |
| Zafirlukast | 0 |
| Zaleplon | 0 |
| Zidovudine | 0 |
| Zileuton | 0 |
| Zinc citrate | 0 |
| Zinc gluconate | 0 |
| Zinc oxide topical | 0 |
| Zinc sulfate | 0 |
| Ziprasidone | 1 |
| Zoledronic acid | 0 |
| Zolmitriptan | 1 |
| Zopiclone | 0 |
| Zuclopenthixol | 1 |
